# HDAC4 mutant represses chondrocyte hypertrophy by locating in the nucleus and attenuates disease progression of posttraumatic osteoarthritis

**DOI:** 10.1186/s12891-021-04947-6

**Published:** 2022-01-03

**Authors:** Xiaodong Gu, Fei Li, Yangyang Gao, Xianda Che, Pengcui Li

**Affiliations:** 1 Department of Orthopaedics, Shanxi Bethune Hospital, Longcheng Road 99, Taiyuan, 030032 China; 2 Department of Orthopaedics, The Second Hospital of Shanxi Medical University, Shanxi Key Laboratory of Bone and Soft Tissue Injury Repair, Wuyi Road 382, Taiyuan, 030001 China

**Keywords:** Histone deacetylase 4 S246/467/632A mutant, Chondrocyte hypertrophy, Nucleus translocation, Osteoarthritis

## Abstract

**Background:**

The aim of this study was to evaluate whether histone deacetylase 4 S246/467/632A mutant (m-HDAC4) has enhanced function at histone deacetylase 4 (HDAC4) to attenuate cartilage degeneration in a rat model of osteoarthritis (OA).

**Methods:**

Chondrocytes were infected with Ad-m-*HDAC4-GFP* or Ad-*HDAC4-GFP* for 24 h, incubated with interleukin-1β (IL-1β 10 ng/mL) for 24 h, and then measured by RT-qPCR. Male Sprague-Dawley rats (*n* = 48) were randomly divided into four groups and transduced with different vectors: ACLT/Ad-*GFP*, ACLT/Ad-*HDAC4-GFP*, ACLT/Ad-m-*HDAC4-GFP,* and sham/Ad-*GFP*. All rats received intra-articular injections 48 h after the operation and every 3 weeks thereafter. Cartilage damage was assessed using radiography and Safranin O staining and quantified using the OARSI score. The hypertrophic and anabolic molecules were detected by immunohistochemistry and RT-qPCR.

**Results:**

M-*HDAC4* decreased the expression levels of *Runx-2*, *Mmp-13*, and C*ol 10a1*, but increased the levels of C*ol 2a1* and *ACAN* more effectively than HDAC4 in the IL-1β-induced chondrocyte OA model; upregulation of *HDAC4* and m*-HDAC4* in the rat OA model suppressed Runx-2 and MMP-13 production, and enhanced Col 2a1 and *ACAN* synthesis. Stronger Safranin O staining was detected in rats treated with m-*HDAC4* than in those treated with *HDAC4*. The resulting OARSI scores were lower in the Ad-m-*HDAC4* group (5.80 ± 0.45) than in the Ad-*HDAC4* group (9.67 ± 1.83, *P* = 0.045). The OARSI scores were highest in rat knees that underwent ACLT treated with Ad-*GFP* control adenovirus vector (14.93 ± 2.14, *P* = 0.019 compared with Ad-*HDAC4* group; *P* = 0.003 compared with Ad-m-*HDAC4* group). Lower Runx-2 and MMP-13 production, and stronger Col 2a1 and ACAN synthesis were detected in rats treated with m-*HDAC4* than in those treated with *HDAC4*.

**Conclusions:**

M-HDAC4 repressed chondrocyte hypertrophy and induced chondrocyte anabolism in the nucleus. M-HDAC4 was more effective in attenuating articular cartilage damage than HDAC4.

## Background

Osteoarthritis (OA) is a major cause of chronic pain and joint dysfunction in the elderly [1]. Currently, there are no effective treatments to delay the progression of OA. The pathological changes in OA include progressive articular cartilage disruption and osteophyte formation [2]. Most OA studies focus on articular cartilage because cartilage damage is the major pathologic feature of OA.

Studies have demonstrated that chondrocyte hypertrophy plays a significant role in the cartilage degeneration of OA. Hypertrophic chondrocytes lose the synthesis ability of type II collagen (Col 2a1) and aggrecan (ACAN) and then synthetize cartilage matrix degrading enzymes. Matrix metalloproteinase-13 (MMP-13) is the main matrix degrading enzyme, contributing to cartilage matrix degradation and cartilage damage [3, 4].

Runx-2, a key regulator of chondrocyte hypertrophy, was increased in OA cartilage. Histone deacetylase 4 (HDAC4) suppresses chondrocyte hypertrophy by repressing the transcriptional activation of *Runx-2*, while HDAC4 is decreased in OA cartilage [5]. A previous study indicated that overexpression of HDAC4 in IL-1β-stimulated chondrocytes decreased the expression levels of *Runx-2*, *Mmp-13*, and upregulation of HDAC4 in articular cartilage attenuated OA progression in a rat OA model [6]. HDAC4 regulates gene expression by locating in the nucleus. However, the transduction of HDAC4 is mainly localized in the cytoplasm [7]. A significant feature of HDAC4 is translocating between the nucleus and cytoplasm of cells. Normally, HDAC4 is located in the cytoplasm, binding to 14-3-3 proteins [8, 9]. When the three serine residues (S246/467/632A) of HDAC4 are dephosphorylated, they detach from the 14-3-3 proteins and are translocated in the nucleus [10]. Data from our laboratory demonstrated that the HDAC4 S246/467/632A mutant (m-HDAC4) loses the ability to bind to 14-3-3 proteins and enters the nucleus to regulate gene expression in chondrocytes [7]. Thus, m-HDAC4, which is mainly located in the nucleus, may have a superior function to wild-type HDAC4 to repress chondrocyte hypertrophy during OA.

In the present study, we established in vitro and in vivo OA models, which were induced by IL-1β stimulation and rat anterior cruciate ligament transection (ACLT), respectively, to investigate the chondroprotective effect of m-HDAC4.

## Methods

This study was approved by the Institutional Animal Welfare Committee of Shanxi Medical University. All methods were carried out in accordance with ARRIVE guidelines.

### Construction and purification of adenoviral vectors

An adenoviral vector encoding m-*HDAC4-GFP*, *HDAC4-GFP* (Ad-m-*HDAC4-GFP*, Ad-*HDAC4-GFP*) were constructed and purified by Genechem company (GCPA0154819, Shanghai, China). Ad-*GFP* is a negative control.

### Culture of rat costal chondrocyte and gene delivery in vitro

Costal chondrocytes were obtained from thorax of newborn Sprague-Dawley (SD) rats as as previously described [11]. Brifely, the ribs were dissected from the rat thorax and predigested with 0.2% collagenase II for 1 h,and further digested with 0.05% collagenase II for 3 h. The chondrocytes were cultured in DEM/F-12 medium (Hyclone, South Logan, UT, USA) containing 10% fetal bovine serum (Gibco, NSW, Australia). At passage 2, chondrocytes were divided into three group and treated with 200 multiplicities of infection (MOI) of Ad*-*m*-HDAC4-GFP*, Ad*-HDAC4-GFP*, or Ad*-GFP* (1 × 10^9^ plaque-forming units [PFUs]/mL) for 24 h. The subcellular localization of HDAC4 and m-HDAC4 was determined by fluorescence microscopy (Leica DM i8, Wetzlar, Germany). The chondrocytes were then treated with 10 ng/mL IL-1β for 24 h (in vitro OA model). Total mRNA was isolated using real-time qPCR.

### Rat ACLT OA model and ad-m-*HDAC4*, ad-*HDAC4* intra-articular injection

Two-month-old male SD rats (*n* = 48) with healthy appearance, appetite and activity were purchased from the Shanxi Medical University Experimental Animal Department, each rat weighing about 230 g. All the experimental procedures were performed in the Shanxi Medical University Experimental Animal Department. During the experiment, all rats were housed in a temperature- and humidity-controlled environment with a 12 h light/12 h dark cycle. The indoor temperature was controlled at 22 °C-26°Cand the humidity was kept at 50-60%. The rats were conventional feeding with standard rat chow. All rats were adapted for 1 week prior to the operation. During operation, the rats were anesthetized by an intraperitoneal injection of 0.3% pentobarbital sodium (1 ml/100 g). ACLT and sham operations were performed on the right rat knees, as described previously [12]. The rats were randomly divided into four groups by random number table (*n* = 12 per group): (1) ACLT+Ad-*GFP*; (2) ACLT+Ad-*HDAC4-GFP*;(3) ACLT+Ad-m-*HDAC4-GFP*;and (4) sham+Ad-*GFP*. We calculated the sample size based on the range of degrees of freedom (DF). The acceptable range of degrees of freedom (DF) for the error term in an analysis of variance (ANOVA) is between 10 to 20. n = DF/k + 1,where k = number of groups, n = number of subjects per group. Therefore, the number of animals in each group ranges from 2 to 4. However, according to our experimental protocol and preliminary experiment, four femoral condyle cartilages were pooled together to achieve the minimum sample size required for RT-qPCR.In order to meet the statistical power, and make the experimental results more reliable, we prepared 3 pooled samples per group, so there were 12 rats in each group. Ad-*HDAC4-GFP*, Ad-m-*HDAC4-GFP*, and Ad-*GFP* were intra-articularly injected 48 h after operation, and every 3 weeks thereafter(1 × 10^9^ PFUs per knee). Every random four rats in each cage were kept and drank freely. All rats were euthanized by intraperitoneal injection of overdose of pentobarbital sodium 2 months after operation. Throughout the study period, the researchers responsible for animal raising were blind to all subsequent operations involving animals, and the researchers conducting the experiment did not know the grouping.

### Radiography

Rat knee anteroposterior and lateral radiographs were taken using a small-animal X-ray radiography system (UltraFocus, Faxitron, Tucson, AZ, USA) to evaluate OA changes 2 months after operation. The images were taken using “Automatic Exposure Control”.

### Histology

The rat right tibial plateaus were harvested and fixed in 10% formalin for 48 h. Samples were immersed in a 10% EDTA solution for decalcification for 6 weeks, the EDTA solution was renewed once a week. Then the tibial plateaus were cut into two approximately equal halves along the frontal plane, and each half was embedded in a paraffin block. Six-μm frontal sections were cut at 0, 200, 400 μm intervals, and Safranin O/Fast Green staining was performed for three sections from each interval. Cartilage lesions were evaluated by two observers following the Osteoarthritis Research Society International (OARSI) grading system, and the scores were averaged for each rat.

### Immunohistochemistry (IHC)

IHC was performed to detect the distribution of Runx-2, MMP-13, and type II collagen in cartilage sections. The knee joint slides were deparaffinized with xylene, rehydrated with ethanol of different concentrations. Endogenous peroxidase was blocked with 3% hydrogen peroxide for 10 min, and then digested with 0.1% trypsin for 30 min at 37 °C. 5% BSA blocking buffer was used to block nonspecific protein binding, and the sections were then incubated with a primary antibody against rat Runx-2 (bs-1134R; Bisso, Beijing, China), MMP-13 (ab39012; Abcam, Cambridge, UK), or type II collagen (ab34712; Abcam, Cambridge, UK) at 4 °C overnight. Thereafter, the sections were treated with horseradish peroxidase (HRP)-conjugated secondary antibody for 30 min at 37 °C and developed using a DAB chromogen. Images were taken with an automatic digital slide scanner (Pannoramic MIDI, 3DHISTECH, Budapest, Hungary).

### Real-time quantitative PCR (RT-qPCR)

Total RNA was isolated from rat femoral condyle cartilage using the TRIzol™ Reagent. Four femoral condyle cartilages were dissected and pooled together, with three pooled cartilages per group. Total RNA was reverse transcribed to complementary DNA (cDNA) using PrimeScript™ RT Master Mix kit (TAKARA, Shiga, Japan), and RT-qPCR was performed using TB Green™ PCR Kit (TAKARA) with a two-step Real-Time PCR System (Applied Biosystems™ QuantStudio™ 6 Flex, Carlsbad, CA, USA). Relative transcript levels were calculated using the 2^-ΔΔCt^ method, as previously described [13]. Primer sequences are listed in Table 1.

**Table 1:** Sequences of primers

Gene	Sequence(5′-3′)
rat *Col2a1*	F: GAGGGCAACAGCAGGTTCAC R: TGTGATCGGTACTCGATGATGG
rat *ACAN*	F: CTGATCCACTGTCCAAGCACCATG R: ATCCACGCCAGGCTCCACTC
rat *Col10a1*	F: GGATGCCTCTTGTCAGTGCTAACC R: TCATAGTGCTGCTGCCTGTTGTAC
rat *MMP-13*	F: AACCAGATGTGGAGTGCCTGATG R:CACATCAGACCAGACCTTGAAGGC
rat *Runx-2*	F: AACAGCAGCAGCAGCAGCAG R: GCACGGAGCACAGGAAGTTGG
*18S rRNA*	F: CGGCTACCACATCCAAGGAA R: GCTGGAATTACCGCGGCT

### Statistical analysis

One-way analysis of variance (ANOVA) was used to analyze the differences in OARSI scores and gene expression levels of *Runx-2*, *MMP-13*, *Col 10a1*, aggrecan (*ACAN)*, *and Col 2a1*. The least significant difference (LSD) multiple comparison test was used for pairwise comparisons following ANOVA. Differences were considered statistically significant at *P* values < 0.05. Statistical analyses were performed using SPSS version 19.0.

## Results

### Adenovirus-mediated transduction of m-HDAC4 located in nucleus and had an enhanced function to inhibit chondrocyte hypertrophy compared with HDAC4

**Fig. 1:**
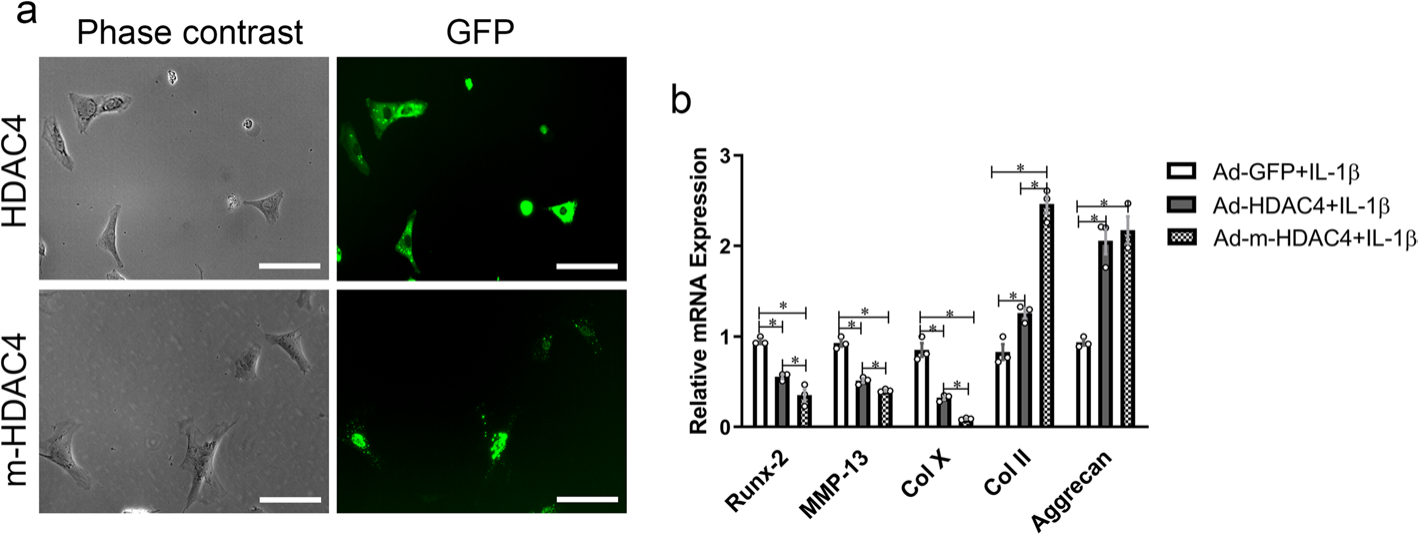
HDAC4 and m-HDAC4 inhibit chondrocyte hypertrophy in vitro OA model. **a** The green fluorescence of m-HDAC4-GFP was located in the nuclei of chondrocytes, while HDAC4-GFP was located in the cytoplasm of cells after infection with adenoviral vectors (MOI:200). Scale bars: 100 μm. **b** RT-qPCR showed that upregulation of m-*HDAC4* decreased the mRNA expression of *Runx-2*, *MMP-13*, and *Col 10a1* and increased the mRNA expression of *Col 2a1* and *ACAN* in rat chondrocytes more effectively than HDAC4.* indicates *P* < 0.05

We used fluorescence microscopy to investigate the subcellular localization of m-HDAC4 and HDAC4. We found that m-HDAC4 was located in the chondrocyte nuclei, while HDAC4 was located in the cytoplasm of chondrocytes, as indicated by green fluorescence at 24 h after adenovirus infection of the cells (Fig. 1a). RT-qPCR was used to detected the mRNA level of hypertrophic and anabolic molecules in chondrocytes. RT-qPCR results showed that, compared with the Ad-*GFP* treated cells (*Runx-2:*0.95 ± 0.04, *Mmp-13:*0.93 ± 0.07*,Col 10a1:* 0.85 ± 0.13, *Col 2a1:*0.83 ± 0.15,*ACAN:*0.94 ± 0.06), *HDAC4* and m-*HDAC4* decreased the expression levels of *Runx-2*(*P* = 0.001 Ad-*HDAC4* compared with Ad-*GFP*; *P* < 0.001 Ad-m-*HDAC4* compared with Ad-*GFP*. *F* = 47.347, df = 8), *Mmp-13*(*P* < 0.001 Ad-*HDAC4* and Ad-m-*HDAC4* compared with Ad-*GFP*. *F* = 112.643, df = 8)*,* and *Col 10a1*(*P* < 0.001 Ad-*HDAC4* and Ad-m-*HDAC4* compared with Ad-*GFP*. *F* = 74.167, df = 8), and they increased the expression levels of *Col 2a1*(*P* = 0.012 Ad-*HDAC4* compared with Ad-*GFP*; *P* < 0.001 Ad-m-*HDAC4* compared with Ad-*GFP*. *F* = 99.810, df = 8) and *ACAN*(*P* = 0.001 Ad-*HDAC4* compared with Ad-*GFP*; *P* < 0.001 Ad-m-*HDAC4* compared with Ad-*GFP*. *F* = 30.561, df = 8). Relative to *HDAC4*, the mRNA levels of *Runx-2*(0.35 ± 0.12), *Mmp-13*(0.40 ± 0.02)*,* and *Col 10a1*(0.09 ± 0.01) in the Ad-m-*HDAC4* treated cells were lower than those in the Ad-*HDAC4* treated cells (*Runx-2:* 0.56 ± 0.04, *P* = 0.018; *Mmp-13:* 0.51 ± 0.04, *P* = 0.025; *Col 10a1:* 0.32 ± 0.04,*P* = 0.011), while the levels of *Col 2a1*(2.46 ± 0.18) were higher than those in the Ad-*HDAC4* treated cells(1.26 ± 0.10, *P* < 0.001) (Fig. 1b).

### Radiology indicated that m-HDAC4 and HDAC4 reduced osteophyte formation in OA

**Fig. 2:**
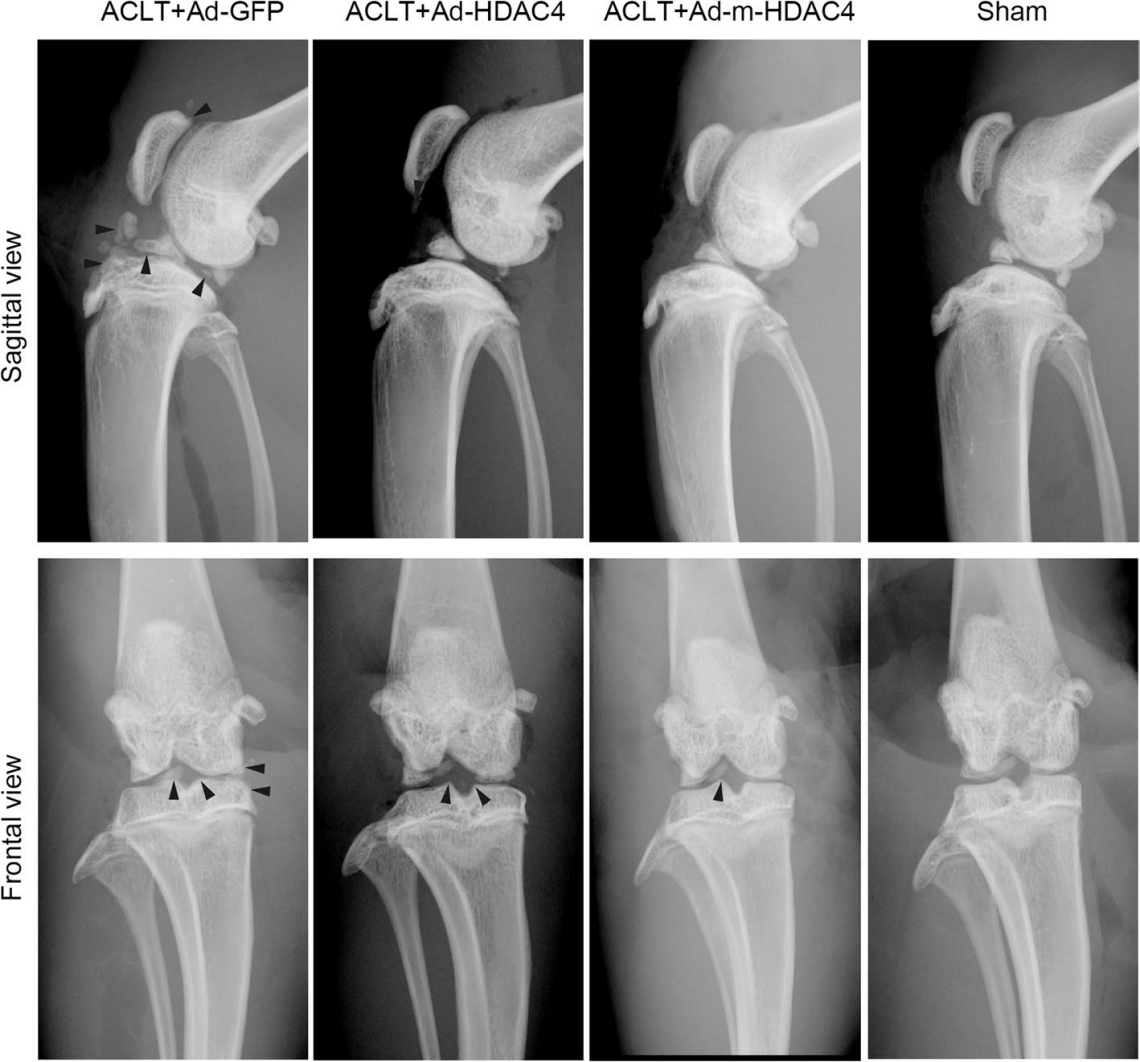
Radiographs of osteophyte formation in the rat knee 2 months after ACLT. The images show slight osteophyte formation along the knee joint margins in the Ad-*HDAC4* and Ad-m-*HDAC4* treated rats; however, the rats than underwent ACLT and treated with Ad-*GFP* demonstrated the most severe change (black arrow heads)

We examined the OA changes of the rat knees by taking X-ray images at 2 months after operation (Fig. 2). The results showed less osteophyte formation along knee joint margins in the Ad-*HDAC4* and Ad-m-*HDAC4* treated rats, and the joint degeneration degree was no obvious difference between the two groups. Osteophyte formation was more evident in the Ad-*GFP* control group.

### Safranin O staining indicated that m-HDAC4 has a stronger chondroprotective effect to attenuate cartilage degeneration, compared with HDAC4 in OA

**Fig. 3:**
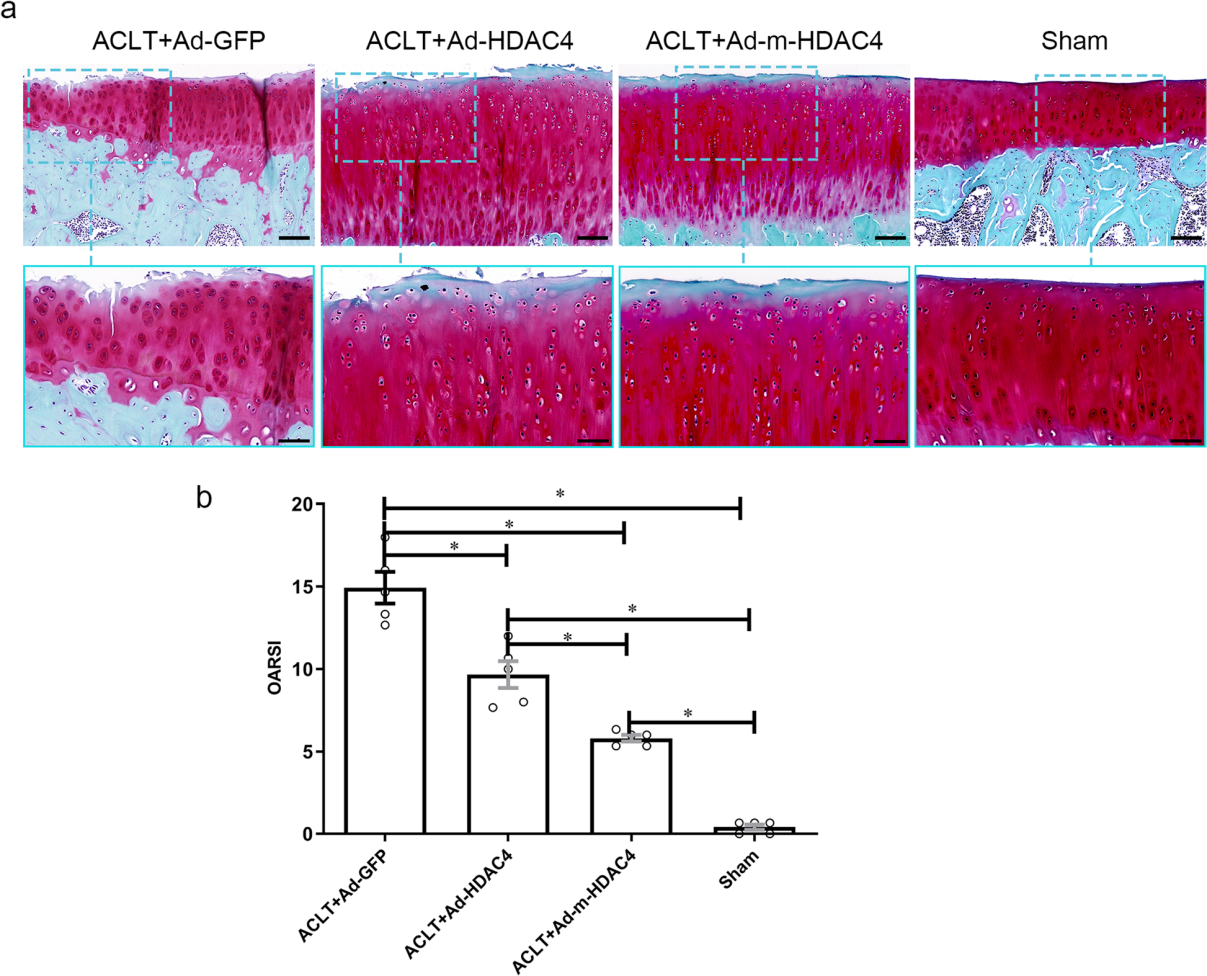
HDAC4 and m-HDAC4 attenuate articular cartilage damage. **a** The Ad-m-*HDAC4* treated rats had stronger Safranin O staining and less-damaged cartilage surface than the Ad-*HDAC4* treated rats. The most severe Safranin O staining lost and cartilage surface damage were detected in rats that underwent ACLT operation and treated with Ad-*GFP*. Top panel scale bars: 100 μm; bottom panel scale bars: 50 μm. **b** OARSI scores were lower in in the Ad-m-*HDAC4* treated rats than in the Ad-*HDAC4* treated rats. The OARSI scores was highest in the ACLT rats and treated with Ad-*GFP* and sham-operated rats had the lowest scores. * indicates *P* < 0.05.

We stained the tibia plateau cartilage with Safranin O/Fast green, which indicated proteoglycan loss and cartilage surface erosion (Fig. 3a). The results showed strong Safranin O staining and intact articular cartilage surface in the Ad-*HDAC4* and Ad-m-*HDAC4* treated rats. The Ad-m-*HDAC4* treated rats showed stronger staining and a less-damaged cartilage surface than Ad-*HDAC4* treated rats. The resulting OARSI scores were lower in the Ad-m-*HDAC4* group (5.80 ± 0.45) than in the Ad-*HDAC4* group (9.67 ± 1.83, *P* = 0.045). The OARSI scores were highest in rat knees that underwent ACLT treated with Ad-*GFP* control adenovirus vector (14.93 ± 2.14, *P* = 0.019 compared with Ad-*HDAC4* group; *P* = 0.003 compared with Ad-m-*HDAC4* group. *F* = 90.748, df = 19) (Fig. 3b).

### IHC showed that m-HDAC4 represses hypertrophy and enhances anabolism of cartilage more effectively than HDAC4

**Fig. 4:**
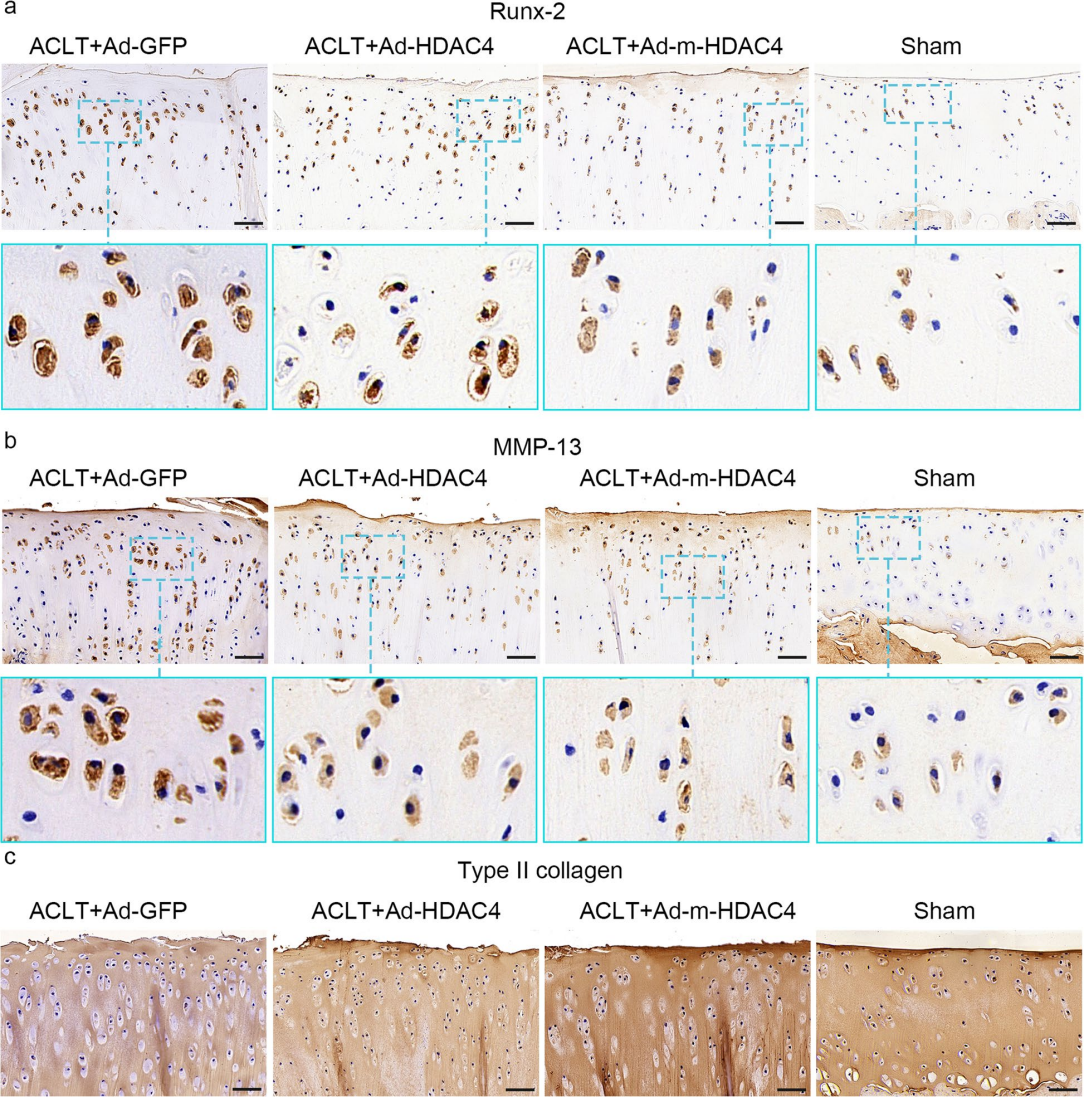
Runx-2 (**a**) and MMP-13 (**b**) IHC staining was increased in rats that underwent ACLT and treated with Ad-*GFP*, but it was lower in the Ad-m-*HDAC4*, Ad-*HDAC4* and sham-operated groups. The staining of Runx-2 and MMP-13 was lower in the Ad-m-*HDAC4* treated rats than in the Ad-*HDAC4* treated rats*.* In contrast, Col 2a1 expression was higher in the Ad-m-*HDAC4* and Ad-*HDAC4* and sham-operated groups than in the Ad-*GFP* treated group. Compared to Ad-*HDAC4* treated rats, Col 2a1 staining was greater in the Ad-m-*HDAC4* treated rats (**c**)

We compared the expression of Runx-2, MMP-13, and Col 2a1 in the four groups using IHC staining of cartilage (Fig. 4). The staining of Runx-2 and MMP-13 was lower in the Ad-*HDAC4* and Ad-m-*HDAC4* treated rats than in the rat that underwent ACLT treated with Ad-*GFP*. Moreover, Runx-2, MMP-13 staining were lower in the Ad-m-*HDAC4* treated rats than in the Ad-*HDAC4* treated rats. In contrast, Col 2a1 expression was higher in the Ad-*HDAC4* and Ad-m-*HDAC4* treated rats than in rats that underwent ACLT operation treated with Ad-*GFP*, and, compared to the Ad-*HDAC4* group, Col 2a1 expression was higher in the Ad-m-*HDAC4* group.

### RT-qPCR indicated that m-HDAC4 represses hypertrophy and enhances anabolism of cartilage more effectively than HDAC4

**Fig. 5:**
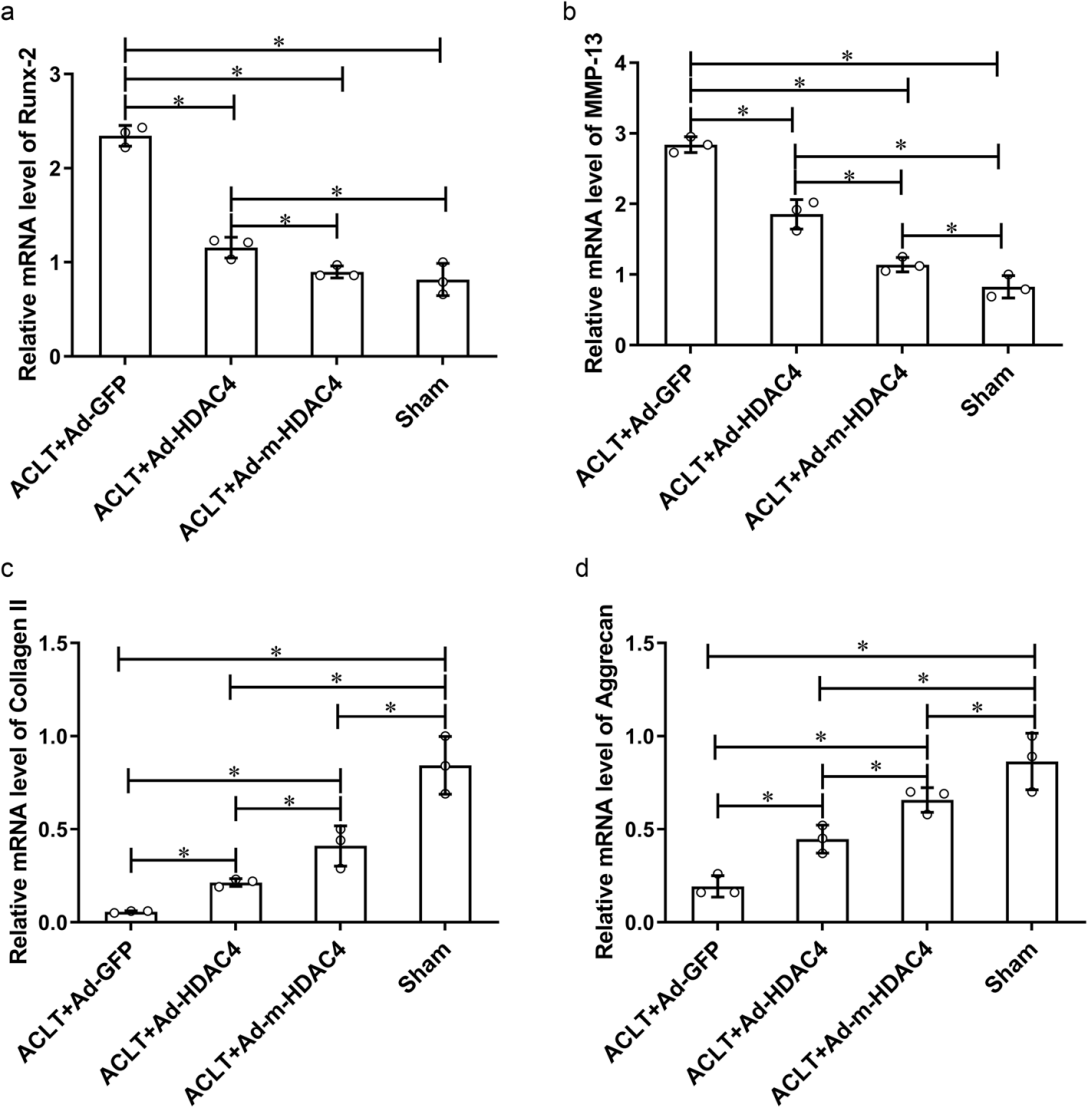
M-HDAC4 repress cartilage hypertrophy and enhance anabolism in rat OA model. **a**, **b** Levels of mRNA for *Runx-2* and *MMP-13* were lower in rats that were treated with Ad-m-*HDAC4* or Ad-*HDAC4* compared to the rats that underwent ACLT and treated with Ad-*GFP*. In addition, these 2 genes were expressed at a lower level in the Ad-m-*HDAC4* treated rats than in the Ad-*HDAC4* treated rats. In contrast, the mRNA levels of *Col 2a1* (**c**) and *ACAN* (**d**) were elevated in rats that were treated with Ad-m-*HDAC4* or Ad-*HDAC4* compared to the rats that underwent ACLT and treated with Ad-*GFP*, and the mRNA expression of *Col 2a1* and *ACAN* were greater in the Ad-m-*HDAC4* treated rats than in the Ad-*HDAC4* treated rats.* indicates *P* < 0.05

RT-qPCR results showed that the mRNA levels of *Runx-2* and *Mmp-13* were lower in the Ad-*HDAC4*(*Runx-2:* 1.16 ± 0.11, *Mmp-13:* 1.85 ± 0.21) and Ad-m-*HDAC4*(*Runx-2:* 0.90 ± 0.06, *Mmp-13:* 1.14 ± 0.10) treated rats than in the rat that underwent ACLT treated with Ad-*GFP* (*Runx-2:* 2.34 ± 0.11, *P* < 0.001 Ad-*HDAC4* and Ad-m-*HDAC4* compared with Ad-*GFP*, *F* = 104.477, df = 11; *Mmp-13:* 2.84 ± 0.11, *P* < 0.001 Ad-*HDAC4* and Ad-m-*HDAC4* compared with Ad-*GFP*, *F* = 105.524, df = 11), while the mRNA levels of *Runx-2* and *Mmp-13* were lower in the Ad-m-*HDAC4* treated rats than in the Ad-*HDAC4* treated rats (*Runx-2: P* = 0.029; *Mmp-13: P* < 0.001). In contrast, the mRNA levels of *Col 2a1* and *ACAN* followed the opposite pattern. The mRNA levels of *Col 2a1* and *ACAN* were higher in the Ad-*HDAC4*(*Col 2a1:* 0.21 ± 0.02, *ACAN:* 0.45 ± 0.08) and Ad-m-*HDAC4*(*Col 2a1:* 0.41 ± 0.11, *ACAN:* 0.66 ± 0.07) treated rats than in the rat that underwent ACLT treated with Ad-*GFP* (*Col 2a1:* 0.06 ± 0.01, *P* < 0.001 Ad-*HDAC4* and Ad-m-*HDAC4* compared with Ad-*GFP*, *F* = 124.582, df = 11; *ACAN:* 0.19 ± 0.06, *P* = 0.012 Ad-*HDAC4* compared with Ad-*GFP*; *P* < 0.001 Ad-m-*HDAC4* compared with Ad-*GFP*, *F* = 27.123, df = 11), while the mRNA levels of *Col 2a1* and *ACAN* were higher in the Ad-m-*HDAC4* treated rats than in the Ad-*HDAC4* treated rats (*Col 2a1: P* = 0.003; *ACAN: P* = 0.027).(Fig. 5).

## Discussion

HDAC4 subcellular translocation plays a vital role in neuronal death [14], myocyte differentiation [15], and chondrocyte differentiation [9]. The primary premise of HDAC4 activation is the nucleus. There are two forms of HDAC4 in cells: one is in the nucleus, which is the activate form, and the other is in the cytoplasm, which has no enzyme activity [16].

HDAC4 is highly important in repressing chondrocyte hypertrophy and regulating growth plate chondrocyte differentiation through relocation of proliferating chondrocytes in the nucleus to prehypertrophic chondrocytes in the cytoplasm. HDAC4 nuclear relocation decreased the gene expression of Runx2, MMP-13, and Col 10a1 and increased the expression of ACAN and Col 2a1 [17]. Our previous study indicated that upregulation of HDAC4 repressed the effect of IL-1β on the expression of catabolic factors in OA chondrocytes and attenuated cartilage degeneration by repressing Runx-2,MMP-13, and Col 10a1 in a rat ACLT OA model [6]. However, the transduction of HDAC4 in chondrocytes is mainly localized in the cytoplasm.

The 14-3-3 protein can promote cytoplasmic localization by binding to HDAC4. The 14-3-3 protein binds to three serine residues (S246, S467, and S632) at the N-terminal of HDAC4 [18], where binding with S246 masks the nuclear localization signal (NLS) and interferes with the binding of importin α and NLS. The 14-3-3 protein also contains a nuclear export signal (NES) when it binds to S467 in the form of a dimer, which is equivalent to providing a NES for HDAC4. Therefore, the 14-3-3 protein can keep HDAC4 in the cytoplasm by inhibiting entry and promoting exit from the nucleus [19–22]. HDAC4 has three serine residues that need to be phosphorylated to bind to the 14-3-3 protein, which requires multiple enzymes [23]. CaMKI phosphorylates S246 and S467, and CaMK II phosphorylates S467 and S632, exposing the 14-3-3 protein anchoring region of HDAC4, and binding to 14-3-3 protein promotes HDAC4 nucleation [24]. In contrast, protein phosphatase 2A (PP2A) can dephosphorylate these HDAC4 binding sites, thus leading HDAC4 into the nucleus, which led to our hypothesis that m-HDAC4, which is mainly located in the nucleus, may induce slower OA progression, compared to HDAC4.

In this study, we constructed an adenoviral vector encoding m-*HDAC4* and *GFP* to infect the rat chondrocytes. The localization of m-HDAC4 was observed by fluorescent microscopy. We observed green fluorescence of m-HDAC4 mainly located in the chondrocyte nuclei, while HDAC4 green fluorescence was mainly located in the cytoplasm. This is consistent with a previous study [7]. We used an in vitro OA model to investigate the protective functions of m-*HDAC4* and *HDAC4* on IL-1β stimulated chondrocytes, and our in vitro results showed that the upregulation of m-*HDAC4* and *HDAC4* ameliorated IL-1β-induced chondrocyte hypertrophy; however, compared with *HDAC4*, m-*HDAC4* had a stronger inhibitory effect on IL-1β-induced chondrocyte catabolism and promoting anabolism.

We used an ACLT OA rat model to investigate the chondroprotective effect of m-HDAC4 on OA in vivo, and our previous study indicated that adenoviral vectors intraarticular injection efficiently transduce *HDAC4* gene in rat articular cartilage [6].

X-ray examination was used to detect osteophyte formation in rat knees. Normally, osteophyte formation is a secondary change in OA, and more osteophytes indicate more severe cartilage damage. Our results showed that the Ad-m-*HDAC4* and Ad-*HDAC4* treated rats had fewer osteophytes in periarticular bone than rats underwent ACLT treated with Ad-*GFP* group. Our radiological results demonstrate that m-*HDAC4* upregulation attenuated joint damage degree.

We futher investigated the therapeutic function of m-*HDAC4* by Safranin O staining. The articular cartilage has stronger staining and a smoother and more-intact cartilage surface in the Ad-m-*HDAC4* group than in the Ad-*HDAC4* group. Then, we used the OARSI score to evaluate rat articular cartilage damage. The OARSI score is a semi-quantitative grading method based on Safranin O staining. The score is positively correlated with articular cartilage damage [25, 26]. We found that the OARSI score is lower in the Ad-m-*HDAC4* group than in the Ad-*HDAC4* group. Thus, m-*HDAC4* transduction had a stronger chondroprotective effect that decreased cartilage aggrecan loss and cartilage damage, compared to HDAC4.

HDAC4 represses the expression of Runx-2 and MMP-13, the key factors of chondrocyte hypertrophy and cartilage matrix degradation, to decrease cartilage damage. Thus, in our in vivo experiment, we measured Runx-2 and MMP-13 using IHC staining and RT-qPCR. The IHC results showed that there were fewer Runx-2 and MMP-13-positive cells in the Ad-m-*HDAC4* treated rats than in the Ad-*HDAC4* treated rats. The RT-qPCR results were consistent with the IHC results, and this inhibitory effect of HDAC4 depends on nucleus relocation [27, 17]. Compared to the cytoplasmic localization of HDAC4, the m-HDAC4 is mainly located in the nucleus, and these results indicate that m-HDAC4 attenuates OA cartilage damage by repressing the expression of Runx-2 and MMP-13 more effectively than HDAC4*.*

In addition, the expression of ACAN and Col 2a1 were upregulated in the Ad-m-*HDAC4* and Ad-*HDAC4* treated rats. Thus, *HDAC4* and m-*HDAC4* upregulation also increased chondrocyte anabolism. This suggests that HDAC4 and its triple mutant may promote chondrocyte proliferation. However, the mechanism of this function is remain unclear and should be investigated in future studies.

The limitations of the study should be mentioned. First, we used rat costal chondrocytes to construct an in vitro OA model to study the effects of M-HDAC4 on chondrocyte anabolism and catabolism. Although studies have shown that costal chondrocytes and articular chondrocytes have the same origin [28] and almost the same biological characteristics and functions [29], knee articular chondrocytes were more appropriate in the study of knee osteoarthritis. Second, this study only evaluated the chondroprotective effect of M-HDAC4, and did not detect the changes of subchondral bone. The changes of subchondral bone were equally important for the evaluation of the treatment effect of osteoarthritis. Third, we use an in vivo OA model of rats, and thus, our findings might not translate directly to humans, as anatomical differences between rats and humans may affect the chondroprotective effect of transgene.

## Conclusions

In conclusion, our study demonstrated that m-HDAC4 and HDAC4 attenuated articular cartilage degeneration by repressing Runx-2 and MMP-13, and by inducing Col 2a1 and ACAN. The m-HDAC4 is more protective than HDAC4.

## Data Availability

The datasets used and/or analysed during the current study are available from the corresponding author on reasonable request.

## Abbreviations

OA: Osteoarthritis
HDAC4: Histone deacetylase 4
m-HDAC4: Histone deacetylase 4 S246/467/632A mutant
Runx-2: Runt-related transcription factor 2
MMP-13: Matrix metalloproteinase 13
Col 2a1: Type II collagen
ACAN: Aggrecan
ACLT: Anterior cruciate ligament transection

## References

[1] Whittaker JL, Truong LK, Dhiman K, Beck C (2021). Osteoarthritis year in review 2020: rehabilitation and outcomes. Osteoarthritis Cartilage.

[2] Bijlsma JW, Berenbaum F, Lafeber FP (2011). Osteoarthritis: an update with relevance for clinical practice. Lancet..

[3] Orfanidou T, Iliopoulos D, Malizos KN, Tsezou A (2009). Involvement of SOX-9 and FGF-23 in RUNX-2 regulation in osteoarthritic chondrocytes. J Cell Mol Med.

[4] Wei F, Zhou J, Wei X, Zhang J, Fleming BC, Terek R, Pei M, Chen Q, Liu T, Wei L (2012). Activation of Indian hedgehog promotes chondrocyte hypertrophy and upregulation of MMP-13 in human osteoarthritic cartilage. Osteoarthr Cartil.

[5] Vega RB, Matsuda K, Oh J, Barbosa AC, Yang X, Meadows E, McAnally J, Pomajzl C, Shelton JM, Richardson JA, Karsenty G, Olson EN (2004). Histone deacetylase 4 controls chondrocyte hypertrophy during skeletogenesis. Cell..

[6] Gu XD, Wei L, Li PC, Che XD, Zhao RP, Han PF, Lu JG, Wei XC (2019). Adenovirus-mediated transduction with histone Deacetylase 4 ameliorates disease progression in an osteoarthritis rat model. Int Immunopharmacol.

[7] Chen C, Wei X, Wang S, Jiao Q, Zhang Y, Du G, Wang X, Wei F, Zhang J, Wei L (2016). Compression regulates gene expression of chondrocytes through HDAC4 nuclear relocation via PP2A-dependent HDAC4 dephosphorylation. Biochim Biophys Acta.

[8] Wang Z, Qin G, Zhao TC (2014). HDAC4: mechanism of regulation and biological functions. Epigenomics..

[9] Guan Y, Chen Q, Yang X, Haines P, Pei M, Terek R, Wei X, Zhao T, Wei L (2012). Subcellular relocation of histone deacetylase 4 regulates growth plate chondrocyte differentiation through Ca2+/calmodulin-dependent kinase IV. Am J Physiol Cell Physiol.

[10] Wang AH, Kruhlak MJ, Wu J, Bertos NR, Vezmar M, Posner BI, Bazett-Jones DP, Yang XJ (2000). Regulation of histone deacetylase 4 by binding of 14-3-3 proteins. Mol Cell Biol.

[11] Li X, Ren X, Li S, Liang J, Zhao X, Wang T, Wang Z (2017). Morphological, Immunocytochemical, and biochemical studies of rat costal chondrocytes exposed to IL-1β and TGF-β1. J Healthc Eng.

[12] Elsaid KA, Zhang L, Waller K, Tofte J, Teeple E, Fleming BC, Jay GD (2012). The impact of forced joint exercise on lubricin biosynthesis from articular cartilage following ACL transection and intra-articular lubricin's effect in exercised joints following ACL transection. Osteoarthr Cartil.

[13] Du G, Zhan H, Ding D, Wang S, Wei X, Wei F, Zhang J, Bilgen B, Reginato AM, Fleming BC, Deng J, Wei L (2016). Abnormal mechanical loading induces cartilage degeneration by accelerating Meniscus hypertrophy and mineralization after ACL injuries in vivo. Am J Sports Med.

[14] Bolger TA, Yao TP (2005). Intracellular trafficking of histone deacetylase 4 regulates neuronal cell death. J Neurosci.

[15] Miska EA, Langley E, Wolf D, Karlsson C, Pines J, Kouzarides T (2001). Differential localization of HDAC4 orchestrates muscle differentiation. Nucleic Acids Res.

[16] Fischle W, Dequiedt F, Hendzel MJ, Guenther MG, Lazar MA, Voelter W, Verdin E (2002). Enzymatic activity associated with class II HDACs is dependent on a multiprotein complex containing HDAC3 and SMRT/N-CoR. Mol Cell.

[17] Chen C, Wei X, Lv Z, Sun X, Wang S, Zhang Y, Jiao Q, Wang X, Li Y, Wei L (2016). Cyclic Equibiaxial tensile strain alters gene expression of chondrocytes via histone Deacetylase 4 shuttling. PLoS One.

[18] Haberland M, Montgomery RL, Olson EN (2009). The many roles of histone deacetylases in development and physiology: implications for disease and therapy. Nat Rev Genet.

[19] Grozinger CM, Schreiber SL (2000). Regulation of histone deacetylase 4 and 5 and transcriptional activity by 14-3-3-dependent cellular localization. Proc Natl Acad Sci U S A.

[20] Lopez-Girona A, Furnari B, Mondesert O, Russell P (1999). Nuclear localization of Cdc25 is regulated by DNA damage and a 14-3-3 protein. Nature..

[21] Rittinger K, Budman J, Xu J, Volinia S, Cantley LC, Smerdon SJ, Gamblin SJ, Yaffe MB (1999). Structural analysis of 14-3-3 phosphopeptide complexes identifies a dual role for the nuclear export signal of 14-3-3 in ligand binding. Mol Cell.

[22] Wang AH, Yang XJ (2001). Histone deacetylase 4 possesses intrinsic nuclear import and export signals. Mol Cell Biol.

[23] Yang XJ, Seto E (2008). The Rpd3/Hda1 family of lysine deacetylases: from bacteria and yeast to mice and men. Nat Rev Mol Cell Biol.

[24] Backs J, Song K, Bezprozvannaya S, Chang S, Olson EN (2006). CaM kinase II selectively signals to histone deacetylase 4 during cardiomyocyte hypertrophy. J Clin Invest.

[25] Pritzker KP, Gay S, Jimenez SA, Ostergaard K, Pelletier JP, Revell PA, Salter D, van den Berg WB (2006). Osteoarthritis cartilage histopathology: grading and staging. Osteoarthr Cartil.

[26] Gerwin N, Bendele AM, Glasson S, Carlson CS (2010). The OARSI histopathology initiative - recommendations for histological assessments of osteoarthritis in the rat. Osteoarthr Cartil.

[27] Nishimori S, Lai F, Shiraishi M, Kobayashi T, Kozhemyakina E, Yao TP, Lassar AB, Kronenberg HM (2019). PTHrP targets HDAC4 and HDAC5 to repress chondrocyte hypertrophy. JCI Insight.

[28] El Sayed K, Haisch A, John T, Marzahn U, Lohan A, Müller RD, Kohl B, Ertel W, Stoelzel K, Schulze-Tanzil G (2010). Heterotopic autologous chondrocyte transplantation--a realistic approach to support articular cartilage repair?. Tissue Eng Part B Rev.

[29] Kitaoka E, Satomura K, Hayashi E, Yamanouchi K, Tobiume S, Kume K, Obinata M, Nagayama M (2001). Establishment and characterization of chondrocyte cell lines from the costal cartilage of SV40 large T antigen transgenic mice. J Cell Biochem.

